# Reproductive health services for Syrian refugees in Zaatri Camp and Irbid City, Hashemite Kingdom of Jordan: an evaluation of the Minimum Initial Services Package

**DOI:** 10.1186/1752-1505-9-S1-S4

**Published:** 2015-02-02

**Authors:** Sandra Krause, Holly Williams, Monica A Onyango, Samira Sami, Wilma Doedens, Noreen Giga, Erin Stone, Barbara Tomczyk

**Affiliations:** 1 Women’s Refugee Commission, 122 East 42nd Street, New York, New York 10168, USA; 2 Centers for Disease Control and Prevention, 1600 Clifton Road, Atlanta, GA 30333, USA; 3 Boston University School of Public Health, 801 Massachusetts Avenue, Boston, MA 02118, USA; 4 United Nations Population Fund, 605 3rd Ave, New York, NY 10158, USA

**Keywords:** Minimum Initial Services Package (MISP); reproductive health; conflict; Jordan; Syrian refugees

## Abstract

**Background:**

The Minimum Initial Services Package (MISP) for reproductive health, a standard of care in humanitarian emergencies, is a coordinated set of priority activities developed to prevent excess morbidity and mortality, particularly among women and girls, which should be implemented at the onset of an emergency. The purpose of the evaluation was to determine the status of MISP implementation for Syrian refugees in Jordan as part of a global evaluation of reproductive health in crises.

**Methods:**

In March 2013, applying a formative evaluation approach 11 key informant interviews, 13 health facility assessments, and focus group discussions (14 groups; 159 participants) were conducted in two Syrian refugee sites in Jordan, Zaatri Camp, and Irbid City, respectively. Information was coded, themes were identified, and relationships between data explored.

**Results:**

Lead health agencies addressed the MISP by securing funding and supplies and establishing reproductive health focal points, services and coordination mechanisms. However, Irbid City was less likely to be included in coordination activities and health facilities reported challenges in human resource capacity. Access to clinical management of rape survivors was limited, and both women and service provider’s knowledge about availability of these services was low. Activities to reduce the transmission of HIV and to prevent excess maternal and newborn morbidity and mortality were available, although some interventions needed strengthening. Some planning for comprehensive reproductive health services, including health indicator collection, was delayed. Contraceptives were available to meet demand. Syndromic treatment of sexually transmitted infections and antiretrovirals for continuing users were not available. In general refugee women and adolescent girls perceived clinical services negatively and complained about the lack of basic necessities.

**Conclusions:**

MISP services and key elements to support implementation were largely in place. Pre-existing Jordanian health infrastructure, prior MISP trainings, dedicated leadership and available funding and supplies facilitated MISP implementation. The lack of a national protocol on clinical management of rape survivors hindered provision of these services, while communities’ lack of information about the health benefits of the services as well as perceived cultural repercussions likely contributed to no recent service uptake from survivors. This information can inform MISP programming in this setting.

## Background

### Minimum initial services package

The need for reproductive health (RH) services is a continuing concern in humanitarian settings, response agencies are increasingly under pressure to document the consequences and outcomes of those programs and services they provide to reduce avoidable morbidity and mortality, particularly among women and girls. Over the years, a variety of claims have been made by the humanitarian response community regarding the direct and indirect benefits of coordinated, high quality RH services, and donors are beginning to ask to see the evidence supporting implementation of those services. The evidence exists but is often of uneven quality, focusing on certain aspects of RH service impacts over others [1].

The Minimum Initial Service Package (MISP) for reproductive health has been a guideline for care in emergencies since the Inter-agency Working Group (IAWG) on Reproductive Health in Crises’ *Reproductive Health in Refugee Situations: An Inter-agency Field Manual* (IAFM) was published in 1996 [2]. The MISP is a coordinated set of priority RH services designed for the onset of an emergency to prevent excess morbidity and mortality, particularly among women and girls. The MISP supports building the foundation for comprehensive RH services that should be initiated as soon as the situation stabilizes (see Table 1). The 1996 IAFM and the MISP standard have undergone revisions in 1999 and 2010. In the 2010 revision of the IAFM, Additional Priorities to the MISP were added to the MISP objectives and priority activities. The Additional Priorities to the MISP include ensuring: contraceptives are available to meet the demand; syndromic treatment of sexually transmitted infections (STIs) is available to patients presenting with symptoms; antiretrovirals are available to continue treatment for people already on antiretrovirals, including for prevention of mother-to-child transmission; and, that culturally appropriate menstrual protection materials are distributed to women and girls. The MISP is also a standard of care in the *Sphere Minimum Standards in Disaster Response* and is therefore part of the standard of care in humanitarian emergencies [3].

**Table 1:** MISP Standard

The major objectives and priority activities that comprise the MISP include [2]: ENSURE the health sector/cluster identifies an agency to lead implementation of the MISP. The lead RH organization: • RH Officer in place • Meetings to discuss RH implementation held • RH Officer reports back to health cluster/sector • RH kits and supplies available and used PREVENT AND MANAGE the consequences of sexual violence: • Protection system in place especially for women and girls • Clinical care available for survivors of rape • Community aware of services REDUCE HIV transmission: • Ensure safe blood transfusion practice • Facilitate and enforce respect for standard precautions • Make free condoms available PREVENT excess maternal and newborn morbidity and mortality: • Emergency obstetric and newborn care services available • 24/7 referral system established • Clean delivery kits provided to birth attendants and visibly pregnant women • Community aware of services PLAN for comprehensive RH services, integrated into primary health care (PHC) • Collect existing background data • Identify suitable sites for future service delivery of comprehensive RH services • Coordinate ordering RH equipment and supplies based on estimated and observed consumption • Assess staff capacity to provide comprehensive RH services and plan for training/retraining of staff ADDITIONAL priority activities • Ensure contraceptives are available to meet the demand • Syndromic treatment of sexually transmitted infections (STIs) is available to patients presenting with symptoms • Antiretrovirals (ARVs) are available to continue treatment for people already on ARVs, including for prevention of mother-to-child transmission. • Ensure that culturally appropriate menstrual protection materials are distributed to women and girls.

To facilitate MISP implementation, the IAWG designed a pre-packaged set of 13 kits containing drugs and supplies for a three-month period. The United Nations Population Fund (UNFPA) leads the development, assembly and delivery of the Inter-agency Reproductive Health Kits contents that are noted in the *Inter-agency Reproductive Health Kits for Crisis Situations*[2].

Previous MISP assessments were conducted in Pakistan (2003), Chad (2004), Indonesia (2005), Kenya (2007) and Haiti (2010) [4-8]. Over the years findings showed gaps in implementation; poor overall coordination including a lack of standard protocols and procedures, lack of donor support, inadequate knowledge of MISP priorities and activities, poor quality and/or availability of referral services, and inadequate monitoring of service delivery. Assessments also revealed variations with regard to the availability of trained staff and supplies needed to prevent excess maternal/neonatal morbidity and mortality, and sexual violence and human immunodeficiency virus (HIV) prevention activities [4-8]. Lastly, findings showed that the MISP remained largely unknown by humanitarian actors for over a decade, but increasing awareness was observed in Haiti [8].

### Syria crisis

Civil unrest in Syria that started in March 2011 resulted in four million persons in need of humanitarian assistance at the time of the assessment, including two million persons who were internally displaced. In addition, just over one million refugees had fled the violence and its aftermath to neighboring countries including: The Hashemite Kingdom of Jordan (Jordan), Lebanon, Iraq, Turkey and countries in North Africa [9]. The social, economic, and health costs of the conflict has disproportionately affected women and girls. An estimated 200,000 pregnant women, including 22,000 women who gave birth every month, and of those almost 15% were at risk of poor outcomes. There were reports that Caesarean sections within Syria had increased from 19% to 45% between 2011 and 2013, respectively [10]. Incidents of gender-based violence, such as sexual harassment and rape, had been reported [11].

### Syrian refugees in Jordan

There were an estimated 355,493 Syrian refugees living in Jordan with 298,025 registered by the United Nations High Commissioner for Refugees (UNHCR) and 57,468 awaiting registration at the time of the assessment. An overwhelming majority of the unregistered refugees were residing in urban areas. The majority (55.2%) of registered refugees were residing in Zaatri camp, with an additional 133,660 refugees residing in urnab areas including 47,087 (15.2%) and 39,339 (13.2%) residing in Irbid and Amman governates, respectively. The largest refugee camp Zaatri hosted 164,365 refugees [12]. As relief agencies ensured that the specific needs of women and girls were factored into humanitarian health response, they relied on the Jordanian Ministry of Health’s (MOH) established guidelines on maternal, newborn care and post abortion care; HIV prevention and treatment; and family planning [13]. Abortion in Jordan is legally permitted to preserve a woman’s physical and mental health or because of fetal impairment [14]. Regarding HIV, Jordan is characterized by a low-prevalence epidemic. Of note is that Jordanian law states that foreigners staying in Jordan beyond three months who are HIV positive can be deported [15]. The reproductive health indicators prior to the crisis in Syria are important to note for agencies in Jordan implementing the MISP. For example, Syria also has low HIV prevalence. A skilled medical staff attends 96% of pregnant women during their births and the Cesarean section rate was 26%. Abortion in Syria is legally permitted only to save a woman’s life. The contraceptive prevalence rate is 54%. Maternal and neonatal mortality rates are 65 deaths per 100,000 live births and 8 deaths per 1000 live births, respectively [16].

### Purpose of the evaluation

This study, one of the six components of the 2012-2014 IAWG global evaluation of RH in humanitarian settings, a decade follow-up to the 2002-2004 IAWG global evaluation, aimed to determine to which extent the MISP was established in an emergency setting. The purpose of this evaluation was to examine to what degree MISP services were in place for Syrian refugees living in Irbid City and Zaatri Camp as an example to highlight factors that both support and hinder the availability and use of MISP services, and to make recommendations towards improved response and scaling-up of services [17].

## Methods

### Site selection

At the time of the evaluation Zaatri Camp had a refugee population of 164,365 and Irbid City 47,087, respectively. Irbid City was included as an urban non-camp refugee site.

### Study design

This was a formative evaluation using three methods; (1) key informant interviews (KIIs), (2) health facility assessment (HFAs), and (3) focus group discussions (FGDs). It was conducted from March 17-22, 2013. The global evaluation team was supported by seven local study staff.

### Domains of evaluation

In order to assess the main variables of interest we examined the domains listed below:

• MISP awareness and knowledge including activities related to MISP response, training of responders in the MISP, awareness of funding allocation for RH including MISP kits, and knowledge of the five MISP objectives.

• Coordination of the MISP including whether regular coordination meetings are held with all relevant stakeholders and how effective coordination meetings were in facilitating MISP coverage.

• Prevent and manage the consequences of sexual violence comprising safe access to and use of health facilities and the availability of clinical care for survivors of sexual violence.

• Reduce HIV transmission including ensuring safe blood transfusion; facilitating and enforcing the implementation of standard precautions at health facilities to prevent the spread of infections; and , making free condoms available.

• Prevent excess maternal and neonatal morbidity and mortality including the availability of emergency obstetric and new born care services and an emergency referral system 24 hours per day 7 days per week; the distribution of clean delivery kits; and, community awareness of existing services.

• Plan for comprehensive RH services, integrated into primary health care including the collection of existing background data; identification of suitable sites for future service delivery of comprehensive RH services; coordination on ordering RH equipment and supplies based on estimated and observed consumption; and, assessing staff capacity to provide comprehensive RH services and planning for training of staff.

• Additional priorities to MISP comprising the availability of contraceptives to meet demand; syndromic treatment of sexually transmitted infections (STIs) to patients presenting with symptoms; antiretroviral medicines to continue treatment for people already on antiretrovirals including for prevention of mother to child transmission; and, culturally appropriate menstrual protection materials for women and girls.

• Assessment of disaster risk reduction and emergency preparedness to determine if these initiatives were undertaken and the extent that the MISP was integrated.

### Sampling

Sampling procedures for KIIs involved a purposeful selection based on a February 2013 mapping of health partners (n=36). Sampling of health facilities included obtaining a list of health facilities that provided RH services in Zaatri Camp (n=15) and Irbid City (n=6). Participants in FGDs were recruited by partner agencies that selected a purposive sample of female youth (18-24 years of age) and older women (aged 25-49 years). In Zaatri Camp, the groups included those that lived near and farther away from health facilities, and newly arrived refugees (arrival within the past two months). In Irbid City, the groups were allocated based on refugee registration status.

### Data collection procedures and analysis

The KIIs questionnaire was modified from one used in past MISP studies [6-8] to integrate the emerging importance of disaster risk reduction and emergency preparedness initiatives and to quantify awareness and knowledge of MISP objectives, activities and the availability of services. Three pilot-tests of the KII tool were undertaken. Invitations to participate in a KII were sent via email to the partners. A member of the study team obtained written consent, conducted the interviews in English with, managers, physicians and nurses and recorded handwritten notes during the interview.

Selected health facilities were visited beforehand by members of the study team to review the HFA evaluation procedure. One relevant staff assisted the assessment teams and oral consent was obtained. The HFA consisted of semi-structured interviews with physicians, managers and nurses conducted in English and use of a standardized check list of equipment and supplies [18].

The FGD tool was modified from a tool used in prior MISP evaluations to accommodate cultural and age appropriate issues among Syrian refugees. The tool was translated into Arabic and back-translated to English. The FGD tool was piloted in Zaatri Camp with two groups of female youth and two groups of older women. FGDs were held in private rooms within health clinics in the camp and in private rooms hosted by local organizations in Irbid City. Verbal informed consent was obtained from all participants.

Data were reviewed across questions and study sections to discern themes and patterns in the information collected in the KIIs. The KIIs interview data were compared across the data from the FGDs to examine similarities and differences. Data from the HFAs were entered into tables and presented as simple numeric data providing descriptive analysis and results; as the number of facilities visited in each setting (Zaatri camp, Irbid city and Mafraq hospital) were too small to use percentages. Quantitative data entry from the HFA was also done in an Excel spreadsheet. Following the completion of the FGDs, the study team member reviewed each question with the facilitator and note takers. At the end of each day, a debriefing was held with all FGD team members to assess any methodological issues, such as translation congruence or questions that were not understood by participants. Notes from the FGDs were translated while in the field. The team coded text into broad themes and sub-topics, and discerned patterns emerging from the information. A question-by-question approach was used to summarize participant comments into multiple themes. During the coding process, data were continuously reviewed, emerging patterns noted and relationships between constructs and themes identified. Data were compared across sites, age groups and registration status. The two study team members who coded the FGD information met routinely to review the themes and gain consensus on interpretation of the results.

### Ethical review

The evaluation protocol was reviewed and cleared by the Centers for Disease Control and Prevention (CDC), UNFPA and United Nations High Commissioner for Refugees (UNHCR) Jordan.

## Results

### Respondents and health facilities

The study team conducted 11 KIIs with agency staff. Five of 15 health facilities run by national and international organizations and militaries were visited in Zaatri Camp. Study sites included three health clinics, one camp hospital, one maternity hospital and the MOH Mafraq referral hospital located outside of Zaatri camp in Mafraq. Six health facilities were visited in Irbid City, two health centers, two clinics, and two hospitals. The team conducted 14 FGDs among refugee women, in Zaatri Camp there were 101 women and in Irbid City there were 58 women, respectively.

### MISP awareness and knowledge

All but one of eleven key informants (KIs) was aware of the MISP, and nearly half knew all five MISP objectives. However, approximately two-thirds of KIs were not aware of the additional priorities of the MISP.

### Coordination of the MISP

Nine KIs reported that UNFPA hosted RH coordination meetings weekly in Zaatri Camp and monthly in Amman. Participants reported that coordination mechanisms, health indicator collection issues (although there was greater emphasis on Zaatri Camp indicators) and MISP implementation was discussed. A KI also said that non-governmental organizations that are not funded are missing from coordination meetings. In addition, several respondents said that RH coordination for urban areas was lagging behind camp coordination because the coordination meetings in Amman tended to focus on the more visible daily refugee influx and refugees concentrated in the camp setting in Zaatri whereas refugees in urban areas, disbursed within host communities were less visible.

The majority of KIs reported that MOH and/or World Health Organization protocols were available to support MISP implementation and funds were available for a MISP response. Three quarters of respondents reported that RH Medical Kits were available and adequate for this response. In both settings, all groups reported that clean home delivery kits were not distributed. One KI explained that given facility-based deliveries were available in Zaatri camp and the urban setting, and the norm among the populations in Jordan and Syria, there was a concern that the distribution of clean delivery kits could encourage home deliveries.

All facilities in Zaatri Camp were open and convenient for adolescent females, but none of the facilities had an appropriate entrance for clients with disabilities. None of the five facilities visited provided RH outreach services. In the FGDs, the majority of women in the Zaatri groups agreed that agencies had not communicated directly with the refugees about the emergency response. Across the groups in Irbid City, most women reported that they were not contacted by agencies and learned about services through their community.

### Prevent and manage the consequences of sexual violence

Seven key informants reported knowledge about measures to prevent sexual violence and treat survivors. However, measures to prevent sexual violence were insufficient and only one site had the human resource capacity and supplies to provide clinical care for rape survivors.

In Zaatri Camp, women expressed concerns about the lack of lighting and their fears of using the toilets at night. In Irbid City, women reported feeling unsafe sending their daughters to school on public buses. Women said that they were fearful of telling their families of sexual violence due to fears of honor killing, or being disowned by family. The women discussed what they perceived as more cases of domestic violence in the camp than what they observed while living in Syria but were fearful of negative consequences if they reported experiencing violence. The women voiced a desire mostly for psychosocial services, in addition to prevention and medical care but were unaware of service availability. Nearly all women across the groups in Irbid City agreed that they would not feel comfortable attending health services for reasons including no benefits from receiving health care and family stigmatization. Additionally, all groups with young women said that they would not tell anyone if they experienced violence. Regarding incidents of sexual violence that are usually reported to UNHCR protection, the Moroccan Field Hospital had not received any sexual violence survivors, although Mafraq Hospital had received one. Treatment and forensic evidence collection was available at Prince Hamza or Mafraq hospitals but they did not have standard protocols. Jordan Health Aid Society (JHAS) clinic was the only facility visited that has a protocol to manage sexual violence survivors in the camp. In Irbid City, there was a formal referral protocol for sexual violence survivors from the health centers to the Family Protection Unit including a standard incident reporting form. Partners stated the MOH was developing a national protocol for clinical management of rape survivors.

### Reduce HIV transmission

Three of nine key informants had essential knowledge on how to reduce HIV transmission. When asked about HIV transmission, all FGDs from Zaatri Camp and five groups in Irbid City stated that they knew about HIV and acquired immunodeficiency syndrome (AIDS). Also, refugee women did not trust the blood supply and had a greater fear of contracting HIV through blood than sexual contact.

Safe blood was available for transfusion in both Zaatri Camp and in Irbid City from a blood bank. Most facilities enforced standard precautions, including use of disposable needles and syringes and sharps disposal boxes. In an event of a health worker’s occupational exposure to HIV, limited occupational post-exposure treatment was available in Amman.

Eight of ten key informants reported that condoms were available through clinics and in women’s safe places. In Zaatri Camp, male condoms were in stock, but female condoms were unavailable. In Irbid City health facilities, most clinics did not supply condoms to non-married women. Men could buy condoms from pharmacies. FGD participants showed very limited knowledge of where they could obtain condoms in Zaatri Camp but participants in Irbid City understood that condoms were available through pharmacies.

### Prevent excess maternal and newborn morbidity and mortality

Approximately half of the key informants could identify all of the priority activities within the objective to prevent maternal and newborn morbidity and mortality. In Zaatri Camp, normal deliveries, basic emergency obstetric care and newborn care functions were conducted at the Gynécologie Sans Frontières maternity clinic. Obstetric emergencies requiring comprehensive emergency obstetric care including post-abortion care and management of newborn complications were referred to the Moroccan Field Hospital. A few women in Zaatri Camp described deterioration in the quality of services over time, including a lack of physical examinations and drugs and unqualified health providers. The deterioration in services may be linked to the large influx of refugees that had been experienced in the months prior to and during the evaluation.

At two Irbid City referral hospitals services for normal deliveries, basic and comprehensive emergency obstetric care, comprehensive abortion care within the law, and post-abortion care were available. FGD participants stated that a UN registration card resulted in free services for pregnant women. Despite free services, women showed reluctance to use them as they were perceived to be “bad” quality due to the lack of privacy and female providers.

A referral system to facilitate transport and communication from the community to health facilities was available in the camp and in Irbid City, with ambulance transportation the most common mode of transport in both settings. Due to traffic congestion, referrals could take 30 minutes or more in the camp, while referrals in Irbid City took 10-45 minutes. In all of the health facilities in Zaatri Camp and Mafraq Hospital, qualified medical personnel were present 24 hours a day, seven days a week but staff complained about an increased case load and insufficient human resources since the onset of the crisis.

### Plan to integrate comprehensive RH services into primary health care

Just over half of the key informants were aware of activities to plan for comprehensive RH services such as assessing and addressing staff capacity to provide comprehensive RH services. Seven of eight respondents reported informing the community of the health benefits to seeking RH services. The majority stated that this was undertaken through health education campaigns. In Zaatri Camp, most reproductive health indicators were collected, but the quality of the indicators was questioned. For example, one report showed a hospital occupancy rate of 120%. Facilities in Irbid City separately reported refugee and non-refugee indicators to the MOH. In terms of planning future sites for delivery of services, UNFPA had recently opened a new maternal and child health center in Zaatri Camp, while planning was also underway to establish more obstetric services for normal deliveries at Primary Health Clinics, at one per 5,000 persons. UNHCR pays health care costs for refugees referred to Mafraq hospital from Zaatri Camp. In Irbid City health facilities, registered refugees did not have to pay for clinical services as they are covered by the MOH. In most government clinics, unregistered refugees, unless they were referred by JHAS and UNHCR covered the cost, paid similar fees to uninsured Jordanians.

In the camp, there were many complaints from FGDs about lack of medications, while in Irbid City, complaints focused on the cost of medications. In Zaatri Camp, requests were made to increase services for special needs populations and vulnerable community members. In Irbid City, the main reasons for not seeking health care among refugees were the disrespect shown to the women by providers, limited or inappropriate medicine and long wait times for care. One KI said that inter-agency service guides on health and protection services had been developed for Syrian refugee-impacted governorates of Jordan. A KII reported that information and education was provided to new arrivals through service booklets, given to JHAS who subsequently distributed them to refugees, including unregistered refugees. In addition, a UNHCR help desk was available.

### Additional priorities of the MISP

An array of family planning methods, including oral contraceptive pills, injectable contraceptives, and intrauterine devices were available. According to Jordanian guidelines emergency contraception can be provided through combined oral contraceptives although a dedicated emergency contraception product was only available for post rape care in one setting. There were provider barriers in access to family planning including emergency contraception. For example, one provider stocked contraceptives but reported that “women did not want them” while another provider reported they would not give emergency contraception to a rape survivor or an unmarried woman. There were cost barriers in the urban context. Although focus group participants expressed a strong need for family planning, half of the participants in Zaatri Camp and almost all in Irbid City were unaware of the locations for free family planning services. Most women in Zaatri Camp and Irbid City mentioned that they would try to self-abort through lifting heavy objects if they had an unwanted pregnancy.

Both providers and service users indicated uneven and inadequate availability of services and supplies related to STIs and HIV, as well as menstrual hygiene. Syndromic management of STIs was not mentioned by representatives of the facilities visited in Zaatri Camp. Most providers said that STI cases were rarely seen. In Irbid City settings providers were not familiar with standard protocols for syndomic management of STIs. None of the facilities at Zaatri Camp provided antiretroviral therapy, including the referral hospital in Mafraq. Those needing antiretroviral therapy were referred to facilities in Amman. It was reported in the FGDs that women in Zaatri Camp received a single distribution of hygiene products upon their arrival but staff at the distribution sites were rude to them. Half of the women had heard about distributions at registration but, when they returned for additional hygiene supplies, they were told that none were available.

### Integration of reproductive health into disaster risk reduction and emergency preparedness

Just over half of KIs reported that there was a national disaster risk reduction agency in Jordan. Mixed responses were received in terms of whether a health risk assessment had been undertaken and whether disaster risk reduction health policies or strategies were in place.

In terms of agency preparedness, approximately two-thirds of respondents reported that their organization undertook preparedness for this crisis. Preparedness trainings included a national training on the MISP in June 2011; the MISP regional training of trainers in Cairo in December 2012; MISP training in Zaatri Camp; and gender-based violence training for police.

Regarding the prepositioning of supplies, while four out of nine KIs reported that RH supplies were procured and pre-positioned, a representative from the agency responsible for this process said that supplies were not pre-positioned.

In summary facilitating factors to MISP implementation are Jordan’s pre-existing health care infrastructure and willingness to address RH among Syrian refugees. Other factors included: the identification of a dedicated agency within the health sector to lead RH coordination; available funding for RH; relative concentration of people in Zaatri Camp; prior MISP training; and, highly skilled and dedicated work force. In contrast, reported barriers to MISP implementation included insufficient funding for the urban response; a lack of female staff; and the absence of a national protocol on clinical management of rape. Other perceived barriers included: limited supplies distribution despite availability; the crisis occurring before Jordan implemented its MISP contingency plan; and the large urban caseload.

## Discussion

### MISP coordination

The importance of coordination in humanitarian crisis has been articulated in global initiatives such as the Interagency Standing Committees humanitarian reform process [19]. The IAWG advocates coordination of RH interventions within the broader humanitarian response to be situated within the health sector. Jordan’s status as an upper middle income country [20], and the regional support it received from other countries to address the Syrian crisis created a solid foundation for the improved MISP policy environment. Appointing a RH lead early in an emergency indicates strong commitment to the issue by the MOH. In comparing urban to camp implementation of MISP, the key difference was that coordination meetings held in Amman, an urban area, were reported to focus on Zaatri camp and had limited attention on Amman or other cities, despite the larger number of refugees in the urban areas. As compared to previous MISP assessments, this MISP assessment shows attention by donors and humanitarian actors to address reproductive health in emergencies as reflected in the leadership by the MOH, UNHCR and UNFPA as well as donor funding for RH and largely sufficient supplies.

### Prevention and response to sexual violence

There appeared to be a lack of priority in the humanitarian response on measures to prevent sexual violence in addition to the challenges to establishing clinical care for rape survivors where the later could be related to the lack of a national clinical management of rape survivor protocol with challenges around the use of emergency contraception and post-exposure prophylaxis. The infrequency of survivors reporting for treatment is possibly related to: Syrian women’s lack of knowledge about the benefits and availability of health care; taboos around talking about sexual violence in the community; and an inadequate number of trained providers/service delivery points. Women are unlikely to weigh the benefits of seeking services against their fears of retribution and cannot make an informed choice about seeking care without knowledge on how medical care can prevent health consequences.

### HIV prevention

In terms of HIV prevention, priority activities were mostly in place, likely due to the existing Jordanian HIV policy and accessible and stocked blood banks. Cultural sensitivities may have inhibited providers from making free condoms visible and readily attainable.

### Prevention of maternal and newborn morbidity and mortality

In order to prevent maternal and newborn morbidity and mortality resulting from obstetric complications, skilled birth attendants, emergency obstetric care and newborn resuscitation should be available and of high quality [21]. These MISP activities were largely in place and facilitated by existing MOH standards, systems and structures for health facility deliveries. In the urban context, the MOH had the benefit of the experience from addressing the needs of the Iraqi refugee population. Despite the availability of services however, many women were displeased with the quality of care that was perceivably impacted by the ongoing surge in refugee influx and the subsequent demands on service providers, as well as the limited number of primary health clinics in Zaatri Camp. A key difference between camp and non-camp based refugees was the use of UNHCR registration card to receive health services outside of the camp, which was repeatedly expressed as a barrier to seeking RH care among refugees. Access to high quality RH services is known to improve health outcomes.

### Information, education and communication about the benefits of seeking care and location of services

Strategies are needed in order to improve acceptance of services and uptake of positive health behaviors. Communication of health information is essential to improve people’s knowledge and acceptance of health services [22]. This form of outreach is important particularly in an emerging crisis setting if prevailing attitudes of the population are negative towards the health care system. Another issue that affects service uptake is stock-out of RH supplies. In both Irbid City and Zaatri Camp delays and gaps persisted in expanding some comprehensive RH services. In light of the ongoing influx of refugees, access to health resources will need to be monitored and maintained despite the changing humanitarian situation. Previous MISP assessments conducted in Haiti (2011) and Indonesia (2005) presented similar gaps in service delivery areas such as care for survivors of sexual violence, in particular, informing communities about the benefits and location of services as well as treatment for rape [8,6].

### Planning for comprehensive reproductive health services

Good collection of RH indicators for monitoring of services brings together relevant partners to ensure that users of health information have access to reliable, authoritative, useable, understandable and comparative information [23]. While the camp and urban contexts are by nature different context, the MOH, UNHCR and UNFPA were all responsible for health including reproductive health. However, in the urban context, health services were largely the responsibility of the MOH with support from local non-governmental organizations whereas services provided in Zaatri camp included external organizations and non-traditional organizations such as the military. A quality health information system takes resources, but it is worth the effort to address obstacles, including poor quality, limited flow, and lack of standardized indicators across agencies. These challenges can be addressed through applying basic surveillance principles and training of staff [24].

### Additional priorities of the MISP

The four additional priorities to the MISP were not very well known by key informants and partially established. The lack of knowledge about the additional priorities to the MISP may be due to the fact that they are relatively new guidelines as they were first put forth in the revised for field testing version of the IAFM in 2010. This evaluation found that some of these services were in place, while others were not. For example, contraceptives were available in both sites, although primarily for married women. Awareness of locations where contraceptives could be obtained was limited. Health care provider biases limited the availability of emergency contraception for Syrian refugees: until a dedicated product is available, providers and refugees can benefit from information and education around the use of oral contraceptive pills as emergency contraception for unprotected intercourse and after rape.

Syndromic treatment of STIs was not available, likely due in part to the absence of a national protocol on treatment of STIs or lack of health seeking for symptoms. In this setting the prevalence of HIV is low but although there was little demand for antiretrovirals there may be a time where this may change and drugs will need to be procured. Lastly, the lack of hygiene, including for menstruation, was upsetting to women and challenging to their sense of dignity. They may have fear due to increased risk of sexual abuse and exploitation as they seek ways to obtain materials.

### Comparison to previous MISP assessments

This MISP assessment showed key informants had more awareness and knowledge about the specific objectives and activities of the MISP as a standard of care in humanitarian emergencies than previous MISP assessments building on the growing awareness noted in the Haiti MISP assessment in 2010 [8]. The greater awareness may be the result of UNFPA and Sexual and Reproductive Health Programme in Crisis and Post-crisis Situations (SPRINT) national and regional training’s on the MISP for Ministry of Health and NGOs over the past several years. Maternal and newborn services were largely in place unlike MISP assessments in Haiti and Pakistan [8,4]. This is likely due to the pre- existing level of maternal and newborn care in Jordan available to urban refugee populations and national and regional partners support of health facilities offering advanced maternal and newborn care in Zaatri camp. Similarly, in this more developed context the availability of safe blood for transfusion and the practice of standard precautions is a standard part of practice pre-crisis while the distribution of condoms is a culturally sensitive issue. However, gaps in prevention of sexual violence and clinical care for survivors of sexual violence are consistent with previous MISP assessments. This could be due to provider’s ongoing lack of commitment to preventing sexual violence and the lack of national protocols for clinical care for survivors of sexual violence. In addition, while key informants in previous MISP assessments reported gaps in funding and supplies as barriers to MISP implementation [4-8], there were very limited to no reports of gaps in funding and supplies to support MISP implementation in Jordan. This could be due to overall funding levels for the Syrian refugee crises and the commitment of MOH, UNFPA and UNHCR to ensure the MISP was integrated in the health sector response [25].

### Limitations

There were several limitations to this evaluation conducted in an ongoing and rapidly evolving emergency that resulted in a large influx of refugees each day. Time and security constraints limited information gathering, especially in the camp. Time constraints for the HFA resulted in the interviewers changing some of the questions and their order to maximize responses from busy informants. For example, the team simply noted that surgery packs for Cesarean sections were available, rather than providing an accurate inventory of all individual items of equipment and supplies. Regarding FGDs, limited time also impacted the team’s ability to probe, which constrained in-depth understanding of some issues. Translation error may also be present, which was countered through daily debriefings with the field team to confirm meanings of words and phrases, and ensure maximum transcription.

## Conclusion

While significant progress has been made in MISP policy and guidelines at the global level, and awareness has grown at the field level, gaps exist in the systematic availability and use of the MISP. The overall availability of MISP services for Syrian refugees in Jordan are consistent with other studies in the IAWG global evaluation showing growing awareness and commitment to the MISP [26,27]. The authors hope that the upward trend to implement the MISP continues in new emergencies, with a focus on enhancing quality of care and an efficient and smooth transition to comprehensive reproductive health services. Still, as is often the case, considerable uncertainty attends any major humanitarian response. Therefore, an important strategy to enhance MISP implementation is to remain focused on the tangible public health lifesaving interventions that women and girls so desperately need in crises.

## List of abbreviations used

AIDS: Acquired immunodeficiency syndrome; CDC: Centers for Disease Control and Prevention; FGD: Focus group discussion; HFA: Health facility assessment; HIV: Human Immunodeficiency Virus; IAWG: Inter-agency Working Group on Reproductive Health in Crises; JHAS: Jordanian Health Aid Society; KII: Key informant interviews; MISP: Minimum initial service package; MOH: Ministry of Health; RH: Reproductive Health; STI: Sexually transmitted infection; UNFPA: United Nations Population Fund; UNHCR: United Nations High Commissioner for Refugees; WRC: Women’s Refugee Commission.

## Competing interests

The authors declare that they have no competing interests.

## Authors’ contributions

BT developed the study protocol and process evaluation methodology with input from SK and MO. BT, HW, SS, MO, WD revised the study tools; SK, HW, SS, MO, WD implemented the study in Jordan; SK and BT led the drafting of the article; SK, BT, HW, SS, MO, WD were co-contributors, with NG and ES contributing to the literature review. All authors reviewed and approved the final text.

## Declarations

This article has been published as part of *Conflict and Health* Volume 9 Supplement 1, 2015: Taking Stock of Reproductive Health in Humanitarian Settings: 2012-2014 Inter-agency Working Group on Reproductive Health in Crises' Global Review. The full contents of the supplement are available online at http://www.conflictandhealth.com/supplements/9/S1. Funding for this supplement was provided by the MacArthur Foundation.
